# Supplementary figures and images for: Cellular and biochemical response to chaperone versus substrate reduction therapies in neuropathic Gaucher disease
Source: PLoS One. 2021 Oct 25;16(10):e0247211. doi: 10.1371/journal.pone.0247211 (PMC8544834; doi:10.1371/journal.pone.0247211)

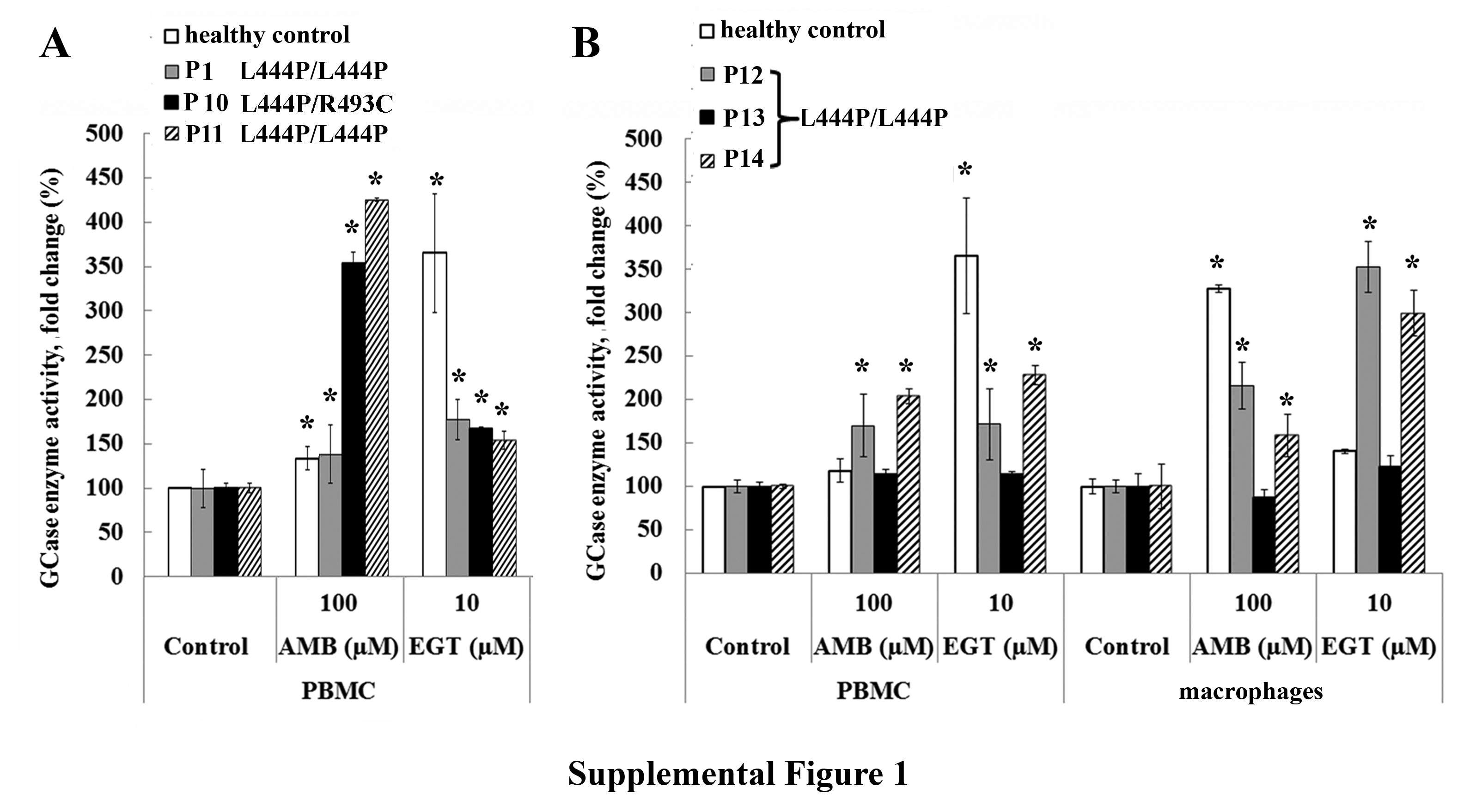

Supplement: S1 Fig — A. PBMC derived from healthy controls (n = 5) and GD3 patients with genotypes L444P/L444P and L444P/R502C were cultured for 5 days in the presence of 100μM AMB and 10 μM EGT. Relative GCase activity was estimated as a fold change towards untreated control. Each bar represents the average +/- STDEV. * p<0.05 compared with an untreated group. B. PBMC and macrophages derived from three GD3 patients with the genotype L444P/L444P, as indicated in the figure, were treated for 5 days in the presence of AMB and EGT. Relative GCase activity was estimated as fold change towards untreated control. Each bar represents the average +/- STDEV. * p<0.05 compared with an untreated group. (TIF) [file pone.0247211.s001.tif]

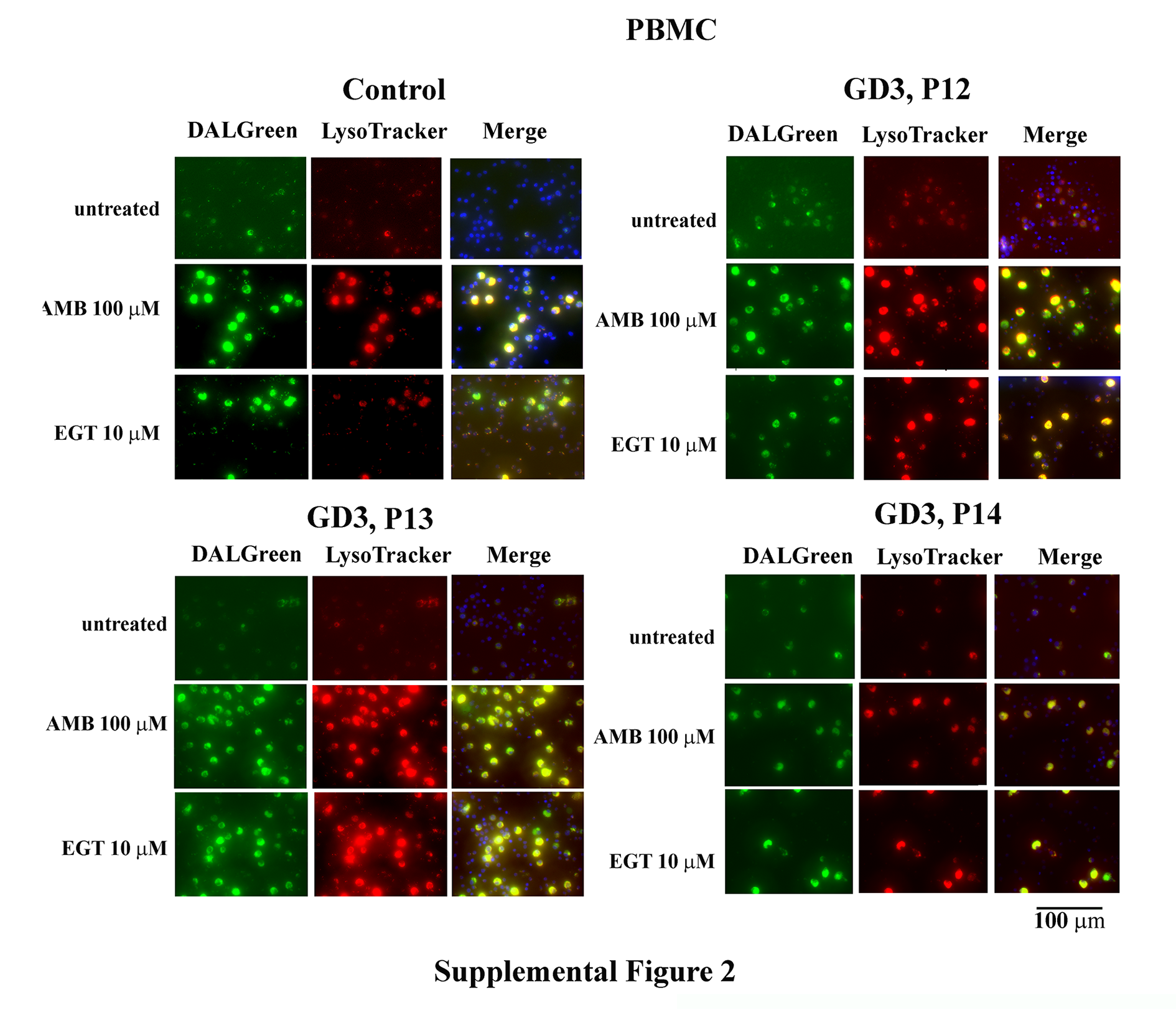

Supplement: S2 Fig — Autophagosome (green, DALGreen) and lysosome (red, LysoTracker) colocalization analysis in PBMC derived from healthy control and GD3 patients (P12, P13, and P14) with the genotype L444P/L444P. (TIF) [file pone.0247211.s002.tif]

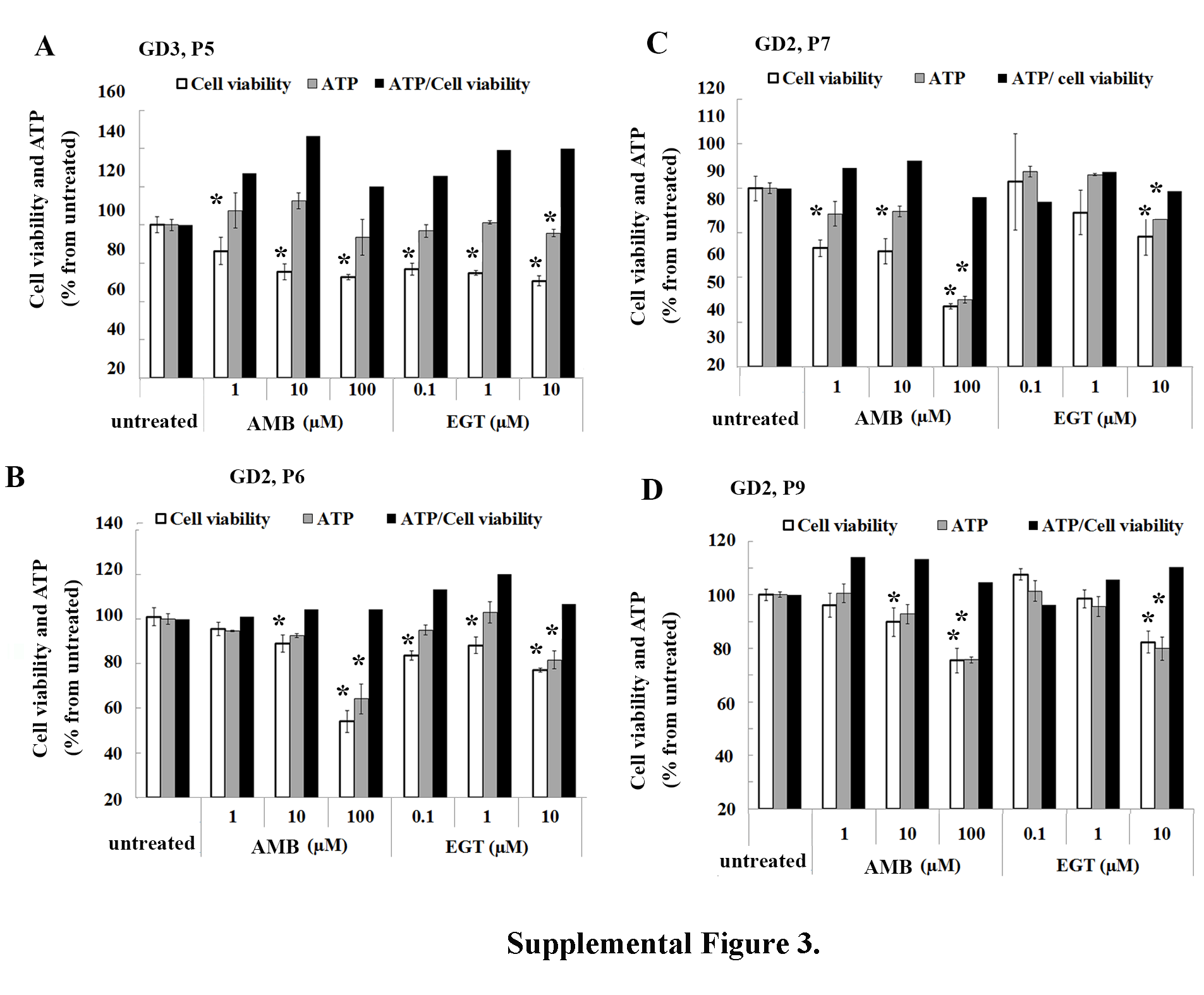

Supplement: S3 Fig — A. P5 fibroblasts from patients with L444P/L444P were treated with 1, 10, 100 μM of AMB and 0.1, 1,10 μM of EGT for 5 days. The CCK-8 cell viability assay, ATP content, and ATP/CCK-8 (cell viability) ratio were analyzed. The cell counting assay, CCK-8, and ATP results were normalized in relationship to the untreated cells. B. P6 fibroblasts with L444P/L444P;RecΔ55;Rec NCiI were treated with 1, 10, 100 μM of AMB and 0.1, 1,10 μM of EGT for 5 days. The CCK-8 cell viability assay, ATP content, and ATP/CCK-8 (cell viability) ratio were analyzed. The cell counting assay, CCK-8, and ATP results were normalized to the untreated cells. C. P7 fibroblasts derived from a patient with L444P/L444P;R495P/R495P;A456P mutation were treated with 1, 10, 100 μM of AMB and 0.1, 1,10 μM of EGT for 5 days. The CCK-8 cell viability assay, ATP content, and ATP/CCK-8 ratio were analyzed. The cell counting assay, CCK-8, and ATP results were normalized to the untreated cells. D. P9 fibroblasts with L444P/D409H were treated with 1, 10, 100 μM of AMB and 0.1, 1,10 μM of EGT for 5 days. The CCK-8 assay, ATP content, and ATP/CCK-8 ratio were analyzed. The results were normalized to the untreated cells. Values are expressed as average ± SEM. * p<0.05 compared with an untreated group. (TIF) [file pone.0247211.s003.tif]

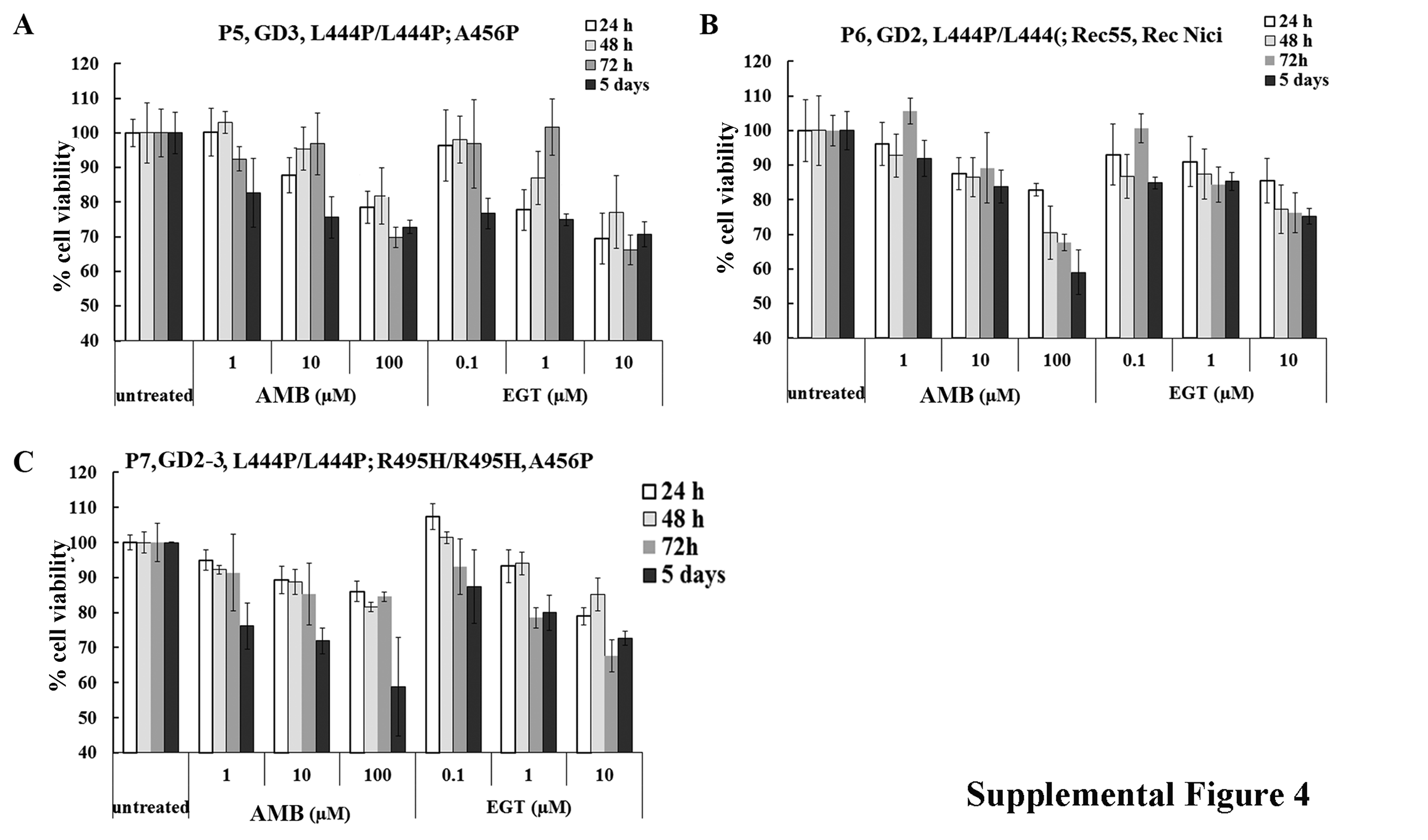

Supplement: S4 Fig — A. P5 fibroblasts derived from patient(s) with the GBA genotype L444P/L444P were treated with AMB and EGT for 24, 48, 72 h, and 5 days. The CCK-8 was analyzed, and results were normalized to the untreated cells. B. P6 fibroblasts were treated with AMB and EGT for 24, 48, 72 h, and 5 days. The CCK-8 assay was measured, and results were normalized to the untreated cells. C. P7 fibroblasts were treated with AMB and EGT for 24, 48, 72 h, and 5 days. The CCK-8 assay was measured, and results were normalized to the untreated cells. Values are expressed as average ±SEM. * p<0.05 compared with an untreated group. (TIF) [file pone.0247211.s004.tif]

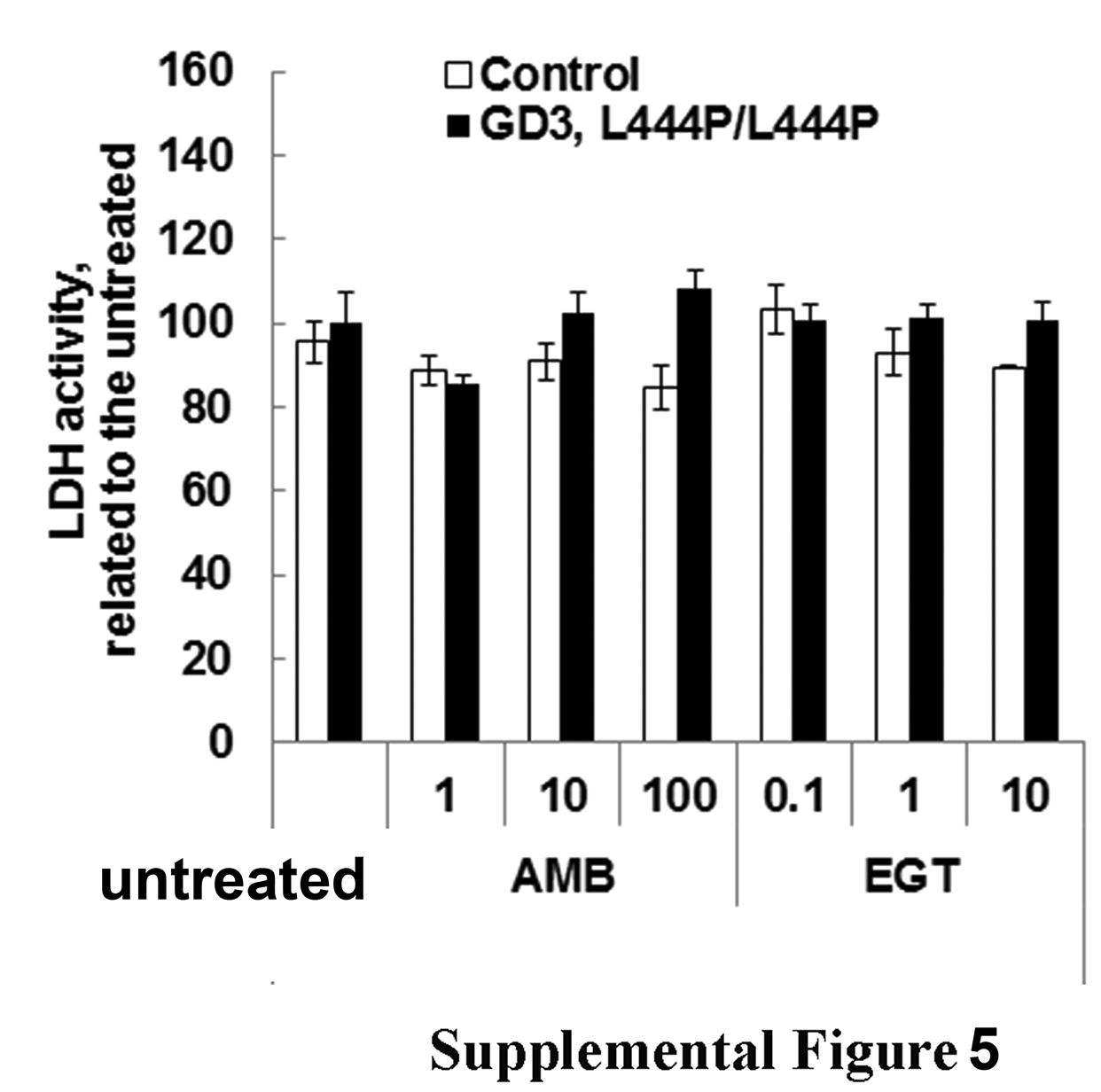

Supplement: S5 Fig — Control and GD3 fibroblasts with L444P/L444P were treated with AMB EGT for 5 days. The LDH assay was analyzed, and results were normalized to the untreated cells. The data represents +/- SEM. (TIF) [file pone.0247211.s005.tif]

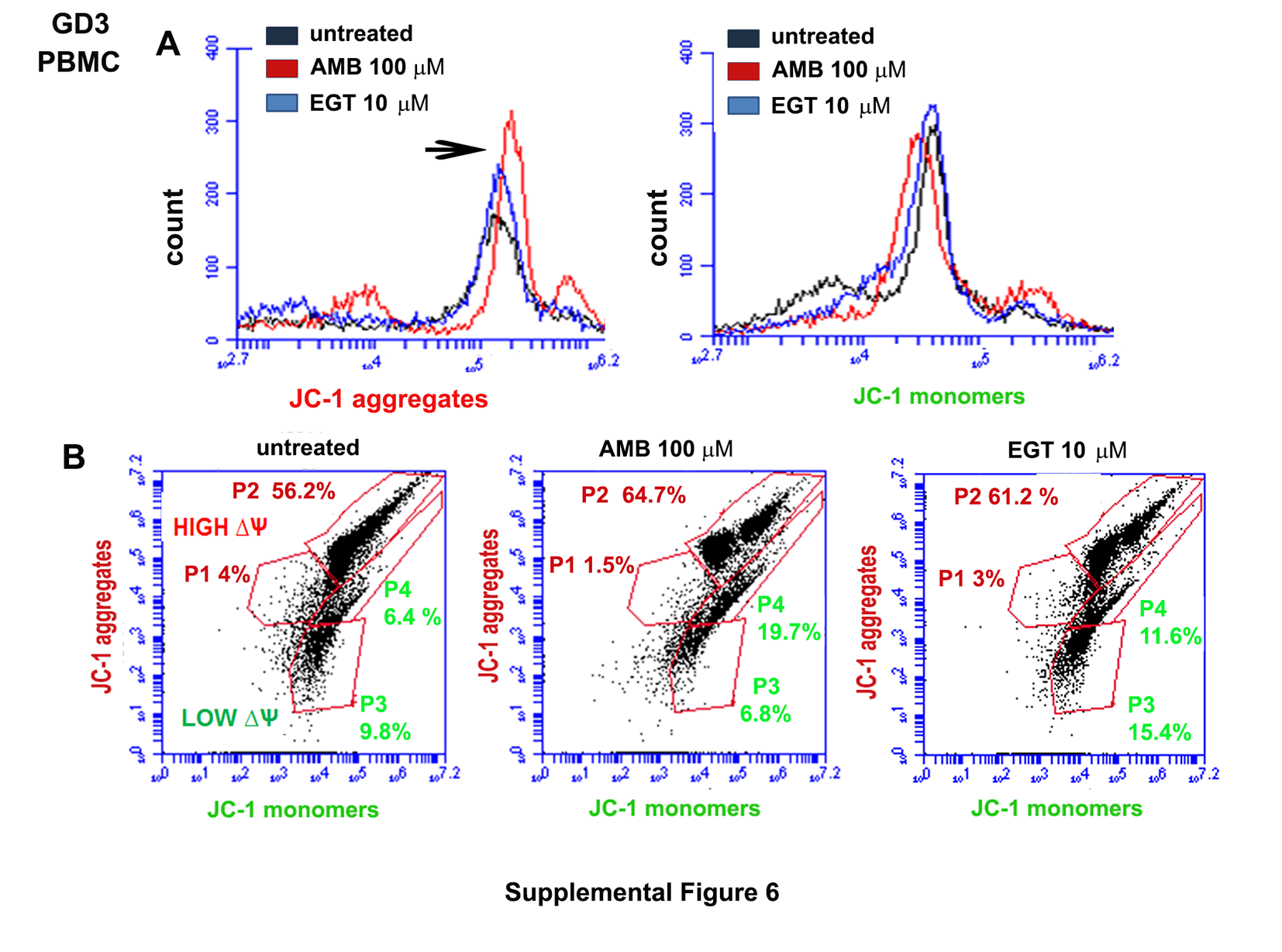

Supplement: S6 Fig — PBMC derived from GD3 patient were treated with AMB and EGT for 5 days. The JC-1 assay was measured Δψ using Flow cytometry. A. Histogram of JC-1 red (left) and green (right) fluorescence intensity of mitochondrial polarization. AMB and EGT induce JC-1 aggregations, as is shown by a shift towards increasing red fluorescent signal. B. Scatterplot shows the identification of cells with polarized (light green) and depolarized (red) mitochondria in GD3 samples. Double-stained cells are present in areas P1, P2, and P4. Cells with preferential green staining are found in area P3. (TIF) [file pone.0247211.s006.tif]
